# Stabilization effect and mechanism of heavy metals by microbial consortium of phosphate-solubilizing bacteria and urease-producing bacteria

**DOI:** 10.3389/fmicb.2025.1525316

**Published:** 2025-02-03

**Authors:** Xuezhe Zhu, Yupin Zhou, Zhenghao Yan, Yongfang Yan, Shuangquan Li, Mingjiao Yu, Xiao Yan, Mingjiang Zhang

**Affiliations:** ^1^National Engineering Research Center for Environment-friendly Metallurgy in Producing Premium Non-ferrous Metals, China GRINM Group Corporation Limited, Beijing, China; ^2^School of Metallurgy, Northeastern University, Shenyang, China; ^3^GRINM Resources and Environment Tech. Co., Ltd., Beijing, China; ^4^General Research Institute for Nonferrous Metals, Beijing, China; ^5^Beijing Engineering Research Center of Strategic Nonferrous Metals Green Manufacturing Technology, Beijing, China; ^6^GRIMAT Engineering Institute Co., Ltd., Beijing, China; ^7^Guobiao (Beijing) Testing & Certification Co., Ltd., China GRINM Group Co., Ltd., Beijing, China; ^8^School of Materials Science and Engineering, Henan University of Science and Technology, Luoyang, China; ^9^Shougang Group Talent Development Institute, Beijing, China

**Keywords:** heavy metal contamination, phosphate-solubilizing bacteria (PSB), urease-producing bacteria (UPB), biological stabilization, biomineralization

## Abstract

**Introduction:**

Stabilization of heavy metals through phosphate-solubilizing bacteria (PSB) induced phosphate precipitation and urease-producing bacteria (UPB) induced carbonate precipitation are promising bioremediation methods. However, little attention has been conducted on the combined action of the above two bioremediations to stabilize heavy metals.

**Methods:**

PSB and UPB were isolated from the environment and their growth characteristics and antagonistic properties were studied. A simulated solution of acidic leachate was prepared based on heavy metal contaminated soil. Microbial consortium of PSB and UPB were constructed for the stabilization of heavy metals by optimizing carbon and nitrogen sources. The microstructural and compositional changes during the biostabilization process were more deeply analyzed using XRD, FT-IR and SEM-EDS.

**Results and discussion:**

The precipitation of heavy metals could be promoted effectively when soluble starch (10.2 g/L) was used as carbon source and urea (7.8 g/L) as nitrogen source. The stabilization rates for Cu, Zn, Cd, and Pb were 98.35, 99.78, 99.09, and 92.26%, respectively. The stabilization rates of the combined action of PSB and UPB were significantly higher than that of the two microorganisms alone. An in-depth analysis showed that the composite metals were precipitated as dense precipitate encased in carbonate and phosphate, and additionally could be stabilized in the form of biosorption. Finally, the stabilization mechanism of heavy metals based on biomineralization and biosorption is proposed. These findings provide new theoretical support for sustainable remediation and management strategies for composite heavy metal polluted areas.

## 1 Introduction

Industrial and agricultural activities contribute to heavy metal emissions, including the mining and smelting of non-ferrous metals, the burning of fossil fuels, the overapplication of fertilizers and the spraying of pesticides on arable land (Malik and Ahmad, 1995; Giller et al., 1998). Among these activities, non-ferrous metal mining and metallurgy is the primary source of heavy metal pollution, accounting for 46.76% of total emissions. Significant accumulations of these migratory heavy metals in smelting sites can leach into underground streams with rainwater, thereby expanding the scope of pollution and affecting the surrounding environment (Xue et al., 2023). The exposure of these heavy metals in the soil will reduce soil fertility and quality (Fei et al., 2019), and seriously affect the growth of plants (Ghori et al., 2019; Wang P. et al., 2019), thus threatening the safety of human life. Therefore, the remediation of soil heavy metals pollution has become an urgent environmental issue. Up to date, many physical, chemical, biological and their combined methods have been developed.

The microbial remediation method solidifies heavy metals through bioconcentration, biosorption, biotransformation, and bioprecipitation (Sun et al., 2021), which has the advantages of wide distribution, rapid treatment of metal pollution, and little impact on soil structure, soil function, and microbial ecological environment, and it has gradually become the mainstream trend (Hou et al., 2023). Most of the current literature focuses on microbial remediation techniques for dealing with single heavy metal contamination (Huang et al., 2023; Si et al., 2023); however, soil heavy metal contamination due to non-ferrous metal smelting is a composite contamination of multiple heavy metals (Oladoye et al., 2022) and multiple microorganisms working synergistically may be able to achieve better stabilization results.

Both phosphate-solubilizing bacteria (PSB) and urease-producing bacteria (UPB) have been widely studied for their excellent remediation properties of heavy metals under aerobic conditions, but their emphases are different. Phosphorus solubilizing microorganisms dissolve insoluble phosphorus into soluble phosphorus through acid production, and the released phosphorus combines with the heavy metals to form insoluble phosphates to solidify the heavy metals, this technique has been used for stabilization of heavy metals such as Cd, Pb, and Cu (Li and Ramakrishna, 2011; Yuan et al., 2017; Li et al., 2018). However, phosphate-solubilizing bacteria dissolve insoluble phosphorus by acid production (Barrow and Lambers, 2022), and excessive action may cause soil acidification and eutrophication. Urease-producing bacteria (UPB) were found to be able to decompose urea into CO_3_^2–^ by releasing urease, which has been used to repair Zn, Cd, and Pb-contaminated soil (Jiang et al., 2019; Zhao et al., 2019; Jalilvand et al., 2020) and improve the quality of vegetables (Wang L. et al., 2019). PSB can increase the risk of heavy metal migration to a certain extent by producing acid to dissolve insoluble phosphorus, and the dissolution of excess ammonia in water after carbonate generation by the UPB causes a decrease in water pH, which leads to the decomposition of some of the carbonate products (Sun et al., 2021). UPB showed a good alkali-producing ability, which can alleviate the risk of heavy metal reverse dissolution caused by the acid production of PSB. In addition, ammonium ions, one of the decomposition products of urea by UPB, can also be used as a nitrogen source for the growth of PSB (Lv et al., 2022), which in turn alleviates the acidification problem caused by the dissolution of ammonium ions in water. The existence of these two mechanisms provides the possibility of PSB-UPB combined solidification of heavy metals. More importantly, adding an appropriate amount of phosphate is conducive to converting carbonate to Fe/Mn oxide-bound fraction (Liao et al., 2023). Therefore, the combination of PSB and UPB has great potential to treat heavy metal pollution in non-ferrous smelting sites and restore autonomous ecological circulation, but there are few relevant studies.

In this study, the optimal nutrient conditions for the combined remediation of heavy metal pollution by PSB and UPB were obtained by optimizing carbon and nitrogen sources, and the composite heavy metal pollution was remediated under the conditions. Then, the remediation products were characterized by SEM-EDS, XRD and FT-IR techniques to reveal the mechanism of the combined remediation of heavy metal pollution by the functional flora based on PSB-UPB.

## 2 Materials and methods

### 2.1 Source of strains

The urease-producing bacteria (*Bacillus aryabhattai* GRMNM-16, *Bacillus megaterium*, GRMNM-17, and *Bacillus* sp., GRMNM-18) and phosphorus-solubilizing bacteria (*Bacillus megaterium*) used in this study were isolated from heavy-metal-contaminated agricultural soils around a smelter in Hunan Province and crop-growing soils in a part of Henan Province.

### 2.2 Soil sources

The test soil was collected from the Laibin smelting plant area in Laibin City, Guangxi Zhuang Autonomous Region, where the average rainfall in the first quarter of 2023 was 172 mm, and the mean rainfall pH was 4.78. Fresh soil samples were collected by picking off the plant residues and gravels, mixing them well, and then air-drying and milling them through a sieve for the determination of the fundamental physicochemical properties of the soil.

The total amount of soil heavy metals Cd, Cu, Zn, and Pb were 360, 700, 10,600, and 3000 mg/kg. The Cd, Cu, and Zn acid leaching concentrations were 1.52, 0.22, and 10.69 mg/L.

### 2.3 Experimental program

The experiment was conducted on a simulated solution of acidic leachate from the test soil. The concentrations of the simulated heavy metal solutions were Cd 5 mg/kg, Pb 5 mg/kg, Cu 5 mg/kg, and Zn 20 mg/kg to investigate the optimal stabilization medium for heavy metals, the stabilization effects of heavy metals, and the mechanism of heavy metals under the joint action of PSB and UPB.

Different carbon and nitrogen sources were selected to explore the growth characteristics, functional expression, and heavy metal stabilization rate of PSB-UPB at a total carbon and nitrogen content of 1.8%, an inoculum size of 7.5%, an incubation temperature of 30°C and a rotational speed of 150 r⋅min^–1^. The culture system’s pH, PO_4_^3–^ and urease activity contents were measured. The total amount of carbon and nitrogen was determined to be 1.8%, where glucose, soluble starch, sodium lactate, and wood chips were the carbon sources for the mixed culture of PSB-UPB; urea, urea + soluble cottonseed cake powder, urea + dried corn syrup, urea + peptone, and urea + yeast extract were the nitrogen sources.

After selecting the best carbon and nitrogen sources, the stabilization effects of the single action of PSB, the single action of UPB and the synergistic action of PSB and UPB on the complex heavy metal pollution were evaluated, respectively.

The experiments were carried out in conical flasks of 300 ml size, three parallel samples were set up for each experimental condition and the supernatant was sampled at regular intervals of 5 ml each time to determine the changes in pH, activity of PSB and UPB and heavy metal concentrations. At the beginning and end of the experiment, the precipitates were taken for interfacial characterization.

### 2.4 Aqueous analysis

The pH was determined by an ion meter (PXSJ-227L, INSEA, Shanghai) using a pH composite electrode (E201-L, INSEA, Shanghai). The activity of PSB and UPB were characterized by the concentration changes of phosphate and ammonium ions (Lv et al., 2022), respectively, which were determined by colorimetry (752-100, Jinghua, Shanghai), and the standard curves for both are shown in Supplementary Figures S1, S2, respectively. The concentrations of Pb, Cu, Cd, Zn were determined by an inductively coupled plasma-emission spectrometer (ICP-OES, Agilent 725, Agilent Technologies Co., Ltd., United States). By comparing the initial concentration with the real-time concentration of heavy metals during stabilization, the stabilization rate can be calculated accordingly.

### 2.5 Interface characterization

At the end of the experiment, the precipitated substrate after the reaction was collected and centrifuged at 4°C and 8,000 rpm (Centrifuge 5810, Eppendorf, Germany). Subsequently, the substrates were frozen in a -80°C ultra-low temperature refrigerator (TDE30086FV-ULTS, Thermo Scientific, United States), and then freeze-dried in a vacuum freeze-dryer (Labconco™ FreeZone™ 2.5L, ThermoFisher, United States). Subsequently, they were sealed and stored and subjected to X-ray diffraction (XRD) (SmartLab, Rigaku, Japan), X-ray photoelectron spectroscopy (XPS) (Escallab, Thermo Fisher, United States), Fourier transform infrared spectroscopy (FT-Infrared), and X-ray photoelectron spectroscopy (XPS) (Escallab, Thermo Fisher, United States) as soon as possible. spectroscopy (FT-IR) (IS50, Nicolet iS50, Thermo Fisher, United States), scanning electron microscope (SEM) (SU8100, Hitachi, Japan), and other interface characterizations.

## 3 Results and discussion

### 3.1 Growth characteristics of phosphate-solubilizing bacteria

**FIGURE 1:**
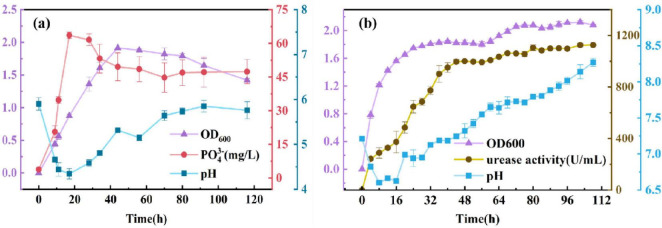
Growth characteristics of **(A)** PSB and **(B)** UPB during pure culture.

To investigate the growth characteristics of PSB, PO_4_^3–^ concentration, and pH in its pure culture of National Botanical Research Institute’s Phosphate (NBRIP) system were determined, and the results are shown in Figure 1A. The concentration of PO_4_^3–^ showed a trend of continuous increase firstly, suggesting that PSB was able to dissolve tricalcium phosphate into soluble phosphorus consistently and steadily during this process. In particular, PSB with a strong capacity of phosphorus dissolution increased to 63.58 mg/L of PO_4_^3–^ in the first 17 h. In addition, Figure 1A also shows that the metabolic activity of PSB can significantly affect the pH of the culture system. The pH decreased from 5.9 to 4.3 in the first 17 h, then slowly rose to stabilize at around 5.8. The initial decrease in pH observed during the growth of the dephosphorylating bacteria is mainly due to the production of metabolites such as organic acids during metabolism by the dephosphorylating bacteria, which can effectively convert insoluble phosphorus into soluble forms of phosphorus and promote the bioavailability of phosphorus. This initial acidification process is a key step in the effective utilization of phosphorus by organisms. With the prolongation of incubation time, the pH value gradually rebounded and eventually stabilized, which may be related to the changes in cellular metabolites and the buffering capacity of the environment. According to the changes of OD_600_ versus culture time, the growth process can be divided into two stages: due to abundant nutrition of systems, the PSB could grow rapidly, and the OD_600_ reach to 1.914 in the first 44 h; then, the population of the PSB showed a slow downward trend from 44 to 116 h.

### 3.2 Growth characteristics of urease-producing bacteria

The three urease-producing bacteria were added to LB liquid medium at a volume ratio of 1%, 1:1:1, respectively. Urease activity pH in its pure culture of LB system was determined, and the results are shown in Figure 1B. The activity of urease showed a steady trend after a continuous increase with the extension of culture time, indicating that UPB could significantly secrete urease in the process. In particular, UPB had a rapid rate of secreting urease in the first 44 h of culture, and urease activity reached 1125 U/mL at 108 h. In addition, Figure 1B also showed that the metabolic activity of UPB could significantly affect the pH of the culture system, which had good alkali production ability. The pH decreased from 7.21 to 6.63 in the first 16 h, then kept increasing to 8.27 at 108 h. According to the changes of OD_600_ versus culture time, the growth process could be divided into two stages: during the culture time of 0–24 h, the UPB could multiply as the nutrient in the medium was rich, reaching the maximum (OD_600_ = 1.75) at 24 h; then, the number of microorganisms showed a stable trend in 24–108 h. Overall, urease activity showed the same trend as OD_600_, and the pH increased as the reaction progressed.

### 3.3 Antagonistic properties

The metabolites of one strain may affect the growth or function of other strains when strains are mixed in culture, thus affecting the function of the whole system. Therefore, examining whether antagonism occurs between strains is a prerequisite for performing mixed culture.

After PSB and UPB were cultured to logarithmic phase, respectively, the two were cross-hatched on the plate containing LB agar medium, and then put into the incubator at 30°C for a period of time and then taken out for observation, and the results were shown in Supplementary Figure S3. The ring of inhibition was not observed on the plate, so it can be assumed that there is no antagonism between the two types of bacteria, and the subsequent mixed culture can be carried out (Bonev et al., 2008).

### 3.4 Enhancement of nutrients in a mixed culture of PSB-UPB

To enhance stabilization rate of Heavy Metals, different carbon sources (glucose, soluble starch, sodium lactate, wood chip and sucrose at 10.2 g/L) and nitrogen sources (urea, urea+soluble cottonseed cake, urea+corn syrupdry powder, urea+peptone and urea+yeast extract at 7.8 g/L) have been examined separately by shake flask method (20°C and 150 rpm agitation for 72 h).

#### 3.4.1 Impact of carbon media composition to growth and heavy metal stabilization rates

**FIGURE 2:**
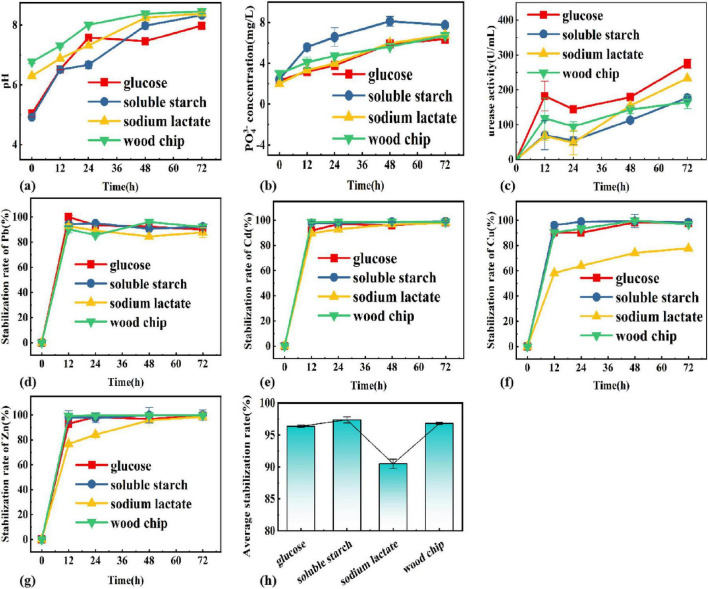
Changes of **(A)** pH, **(B)** PO_4_^3^**^–^**, **(C)** enzyme activity, **(D)** Pb, **(E)** Cu, **(F)** Cd, **(G)** Zn, **(H)** average stabilization rate in different carbon sources systems.

The impact of different carbon sources on microbial growth and heavy metal solidification rate were investigated to optimize the medium composition and the results are shown in Figure 2.

The pH, soluble phosphorus concentration, and urease activity were explored under different carbon source systems as shown in Figure 2 The different carbon sources contributed to the increase in pH, where the highest pH was reached at 8.46 for the system with wood chips on the third day, and the highest increase was for the system with soluble starch from 4.92 to 8.33. This indicates that the functional colony was able to coexist in its natural environment using wood chips to obtain the required carbon sources. For the four types of carbon sources mentioned above, it was the system of soluble starch that had the greatest effect on pH in the growth activity of PSB-UPB. The fastest increase in PO_4_^3–^ concentration was in the soluble starch system, with the highest dissolved phosphorus reaching 7.5 mg/L, indicating that the use of soluble starch as a carbon source is most favorable for the growth of PSB, and the urease activity continued to increase with time, with the highest urease activity in the system using glucose as a carbon source, followed by sodium lactate, soluble starch, and wood chips.

To identify an ideal carbon source for maximizing the stabilization rate, PSB and UPB were grown 1:1 in composite heavy metal solutions with different carbon sources with different carbon sources (soluble starch, glucose, wood chip, sodium lactate, and glucose). Figure 2 showed the pattern of the stabilization rate of each heavy metal with time for different carbon source types, and the results show that the stabilization rate increases with time. Apart from sodium lactate, good stabilization was established in the system with glucose, soluble starch, and wood chips as the only carbon source. For the average stabilization rate at 72 h, soluble starch > glucose > wood chips > sodium lactate, and the average stabilization rate reached 97.37% in the system with soluble starch, and 92.26, 99.78, 99.09, and 92.26% for Cu, Zn, Cd, and Pb, respectively. Meanwhile, the pH increase and soluble phosphorus content of the soluble starch system were also higher than those of other systems, indicating that it is favorable for the co-growth of urease-producing bacteria and when the carbon source is soluble starch. Thus, soluble starch was selected as the best carbon source.

#### 3.4.2 Impact of nitrogen media composition to growth and heavy metal stabilization rates

**FIGURE 3:**
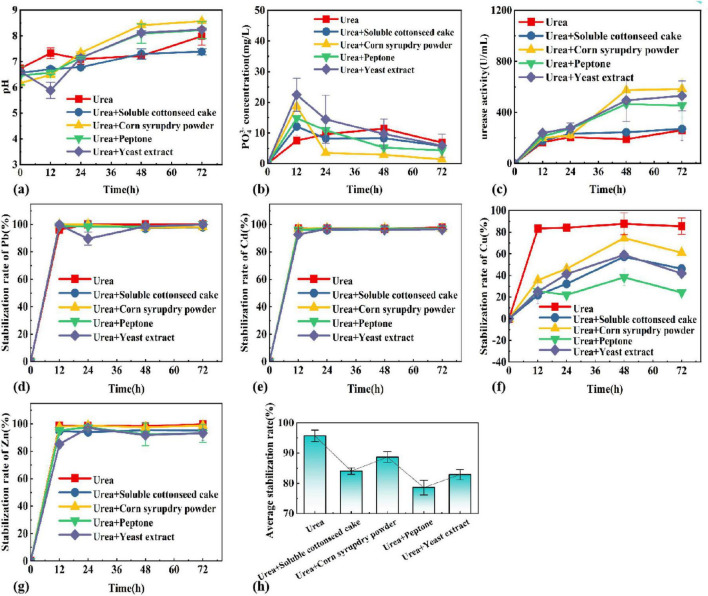
Changes of **(A)** pH, **(B)** PO_4_^3^**^–^**, **(C)** enzyme activity, **(D)** Pb, **(E)** Cd, **(F)** Cu, **(G)** Zn, **(H)** average stabilization rate in different nitrogen sources systems.

Different types of nitrogen sources were selected and the optimal nitrogen source was determined by a combination of pH, PO_4_^3–^ concentration, urease activity, and stabilization rate. Figure 3 shows the pattern of pH, PO_4_^3–^ concentration, urease activity and stabilization rate of each heavy metal with time in different composite nitrogen source systems. The results showed that the corn syrup dry powder could promote the secretion of urease and keep high urease activity in the late stage of the reaction, and the change of pH value was closely linked to the urease activity. The corn syrup dry powder has the potential to function as both a carbon and a nitrogen source. The pH increased as the reaction progressed, and the system with urea + corn syrup dry powder had the highest pH of 8.57 at 72 h of reaction, and the rest of the nitrogen source systems all reached about 8. At the same time, the urease activity of the urea + corn syrup dry powder system was at the highest value, which indicates that pH has a positive correlation with urease activity. However, for the nitrogen source system with the highest pH and urease activity, the PO_4_^3–^ concentration was the lowest at 72 h. Soluble cottonseed cake powder could promote the phosphorus-solubilizing ability of phosphate-solubilizing bacteria in a short time. Still, in the long term, the nitrogen source system of urea only was more favorable to the release of soluble phosphorus. In addition, it has been shown that the addition of urea is beneficial in promoting carbonate precipitate from UPB (Lyu et al., 2022). The stabilization rate of heavy metals increased with the increase of time, where the average size of stabilization rate at 72 h was, urea > urea + corn syrup dry powder > urea + soluble cottonseed cake powder > urea + yeast extract > urea + peptone, and the average stabilization rate of urea system reached 96.64% at 72 h of reaction. Cu, Zn, Cd, and Pb cured at 99.65, 95.08, 98.71, and 93.12%, respectively, so urea was selected as the best nitrogen source.

### 3.5 Evaluation of synergistic stabilization effect

**FIGURE 4:**
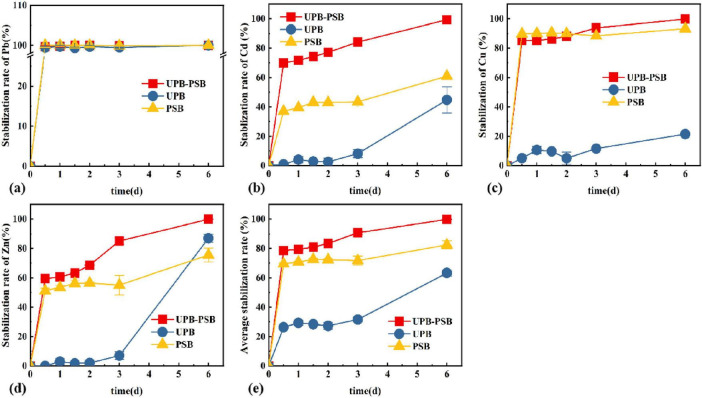
Changes of **(A)** Pb, **(B)** Cd, **(C)** Cu, **(D)** Zn, **(E)** average stabilization rate in different functional bacteria.

The stabilization study of the composite heavy metal solution was carried out with different functional bacteria, respectively, and the results are shown in Figure 4. The stabilization rate increased with the increase of reaction time, and under the action of three different functional bacteria, the stabilization effect of UPB was weaker for Cu and Cd, and better for Pb and Zn, which reached 21.55, 44.88, 100, and 86.99%, respectively, at the 6th day of the reaction. The PSB had a certain degree of stabilization effect on the four heavy metals, but UPB-PSB synergistic stabilization rate was higher than the other restoration groups, with 99.81, 100, 99.35, and 99.87% for Cu, Pb, Cd, and Zn, respectively, at 6 days of restoration.

Through the optimization experiment of carbon source and nitrogen source condition factors, the optimal culture conditions were finally obtained at the soluble starch 10.8 g/L, urea 7.2 g/L, medium conditions to achieve the maximum average stabilization rate.

### 3.6 Microstructure and composition analysis of precipitate

#### 3.6.1 Elemental constitute of precipitated products

**FIGURE 5:**
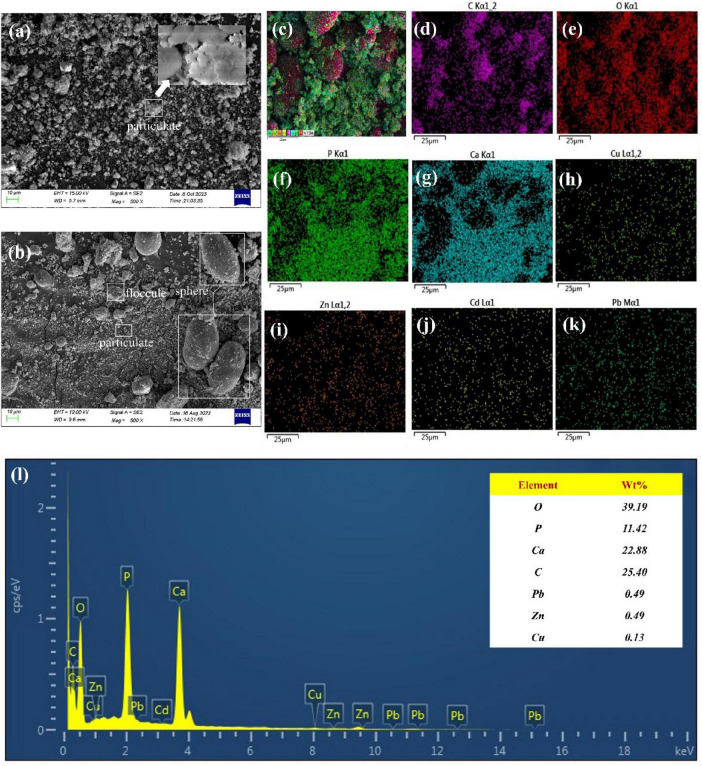
SEM micrographs and EDS analysis of samples **(A)** pre-reaction sediment, **(B)** post-reaction sediment; EDS mapping of **(C)** total elements, **(D)** C, **(E)** O, **(F)** P, **(G)** Ca, **(H)** Cu, **(I)** Zn, **(J)** Cd and **(K)** Pb; **(L)** EDS of post-reaction sediment.

Scanning electron microscopy analysis of the sediment before and after the reaction revealed the results as shown in Figures 5A,B. The precipitate before the reaction showed a uniformly distributed agglomerated accumulation of particulate matter, and magnification revealed that the particulate matter consisted of flaky powders. Figure 5B illustrates the SEM image of the reacted precipitate, the precipitate consists of spherical precipitate, a dense solidified layer, and fine particles, and the surface of the precipitate is tightly structured, and smooth, with small cracks, which is similar in shape to carbonate in the product of the MICP technique (Liu et al., 2020). To further understand the types of elements in the precipitate and their distribution patterns, the products were analyzed by surface energy spectroscopy and surface scanning, respectively, and the results are shown in Figures 5C–K. Figure 5L shows the energy spectrum, indicating that nutrients such as O, P, Ca, and C were detected in the precipitated products, in addition to the presence of the target heavy metal elements such as Pb, Zn, and Cu, which accounted for 0.49%, 0.49%, and 0.13%, respectively. Figures 5H–K are plot of the surface energy spectrum, the presence of the target elements Cu, Zn, Cd, and Pb was detected in the precipitate. The elements C and O were concentrated on the spherical precipitate. In contrast, the elements P and Ca were consistent with the fine-grained division, which, combined with the SEM analysis, were mainly the Ca_3_(PO_4_)_2_ that had not been dissolved yet. Figures 5H–K show Cu, Zn, Cd, and Pb distribution patterns in the sediment, respectively. It was found that the above four types of metals were all relatively well precipitated in the cured product, and their concentration in the precipitate mid-face sweep was lower than the elemental concentration of C, O, P, Ca, etc., in the medium due to the lower concentration of heavy metals in the simulated wastewater solution. Wu et al. (2020) reported that CaCO_3_ products produced by urease-producing bacteria are mainly spherical precipitate. As for the precipitates after the reaction, it is tentatively suggested that dense material represented by spherical precipitate is the main solidification product.

**FIGURE 6:**
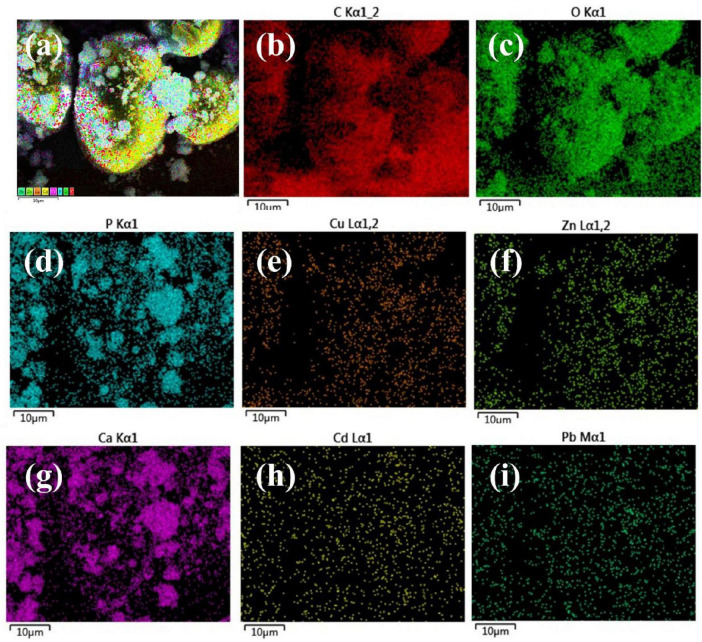
EDS analysis of samples **(a)** total elements, **(b)** C, **(c)** O, **(d)** P, **(e)** Cu, **(f)** Zn, **(g)** Ca, **(h)** Cd and **(i)** Pb.

According to the results of the total sediment surface sweep and the elemental content detection analysis, the dense product, typified by the spherical precipitate, is the main carbonate-based stabilization product. Therefore, we carried out the major elemental composition distribution surface sweep analysis of the spherical precipitate, and the results are shown in Figure 6. C, O, P, and Ca are the main constituents of the precipitate, with P and Ca following the same distribution pattern as the fine grains. In addition, the target elements Cu, Zn, Cd, and Pb were all detected in the sediments, with Cd and Pb distributed relatively uniformly in the detection field of view, and Cu and Zn mainly concentrated on the spherical precipitate. The sediments are predominantly in the form of carbonate and phosphate compositions, and the morphology of the products resembles that of stable calcite (Burbank et al., 2012).

#### 3.6.2 Characterization of elemental properties of precipitated products

**FIGURE 7:**
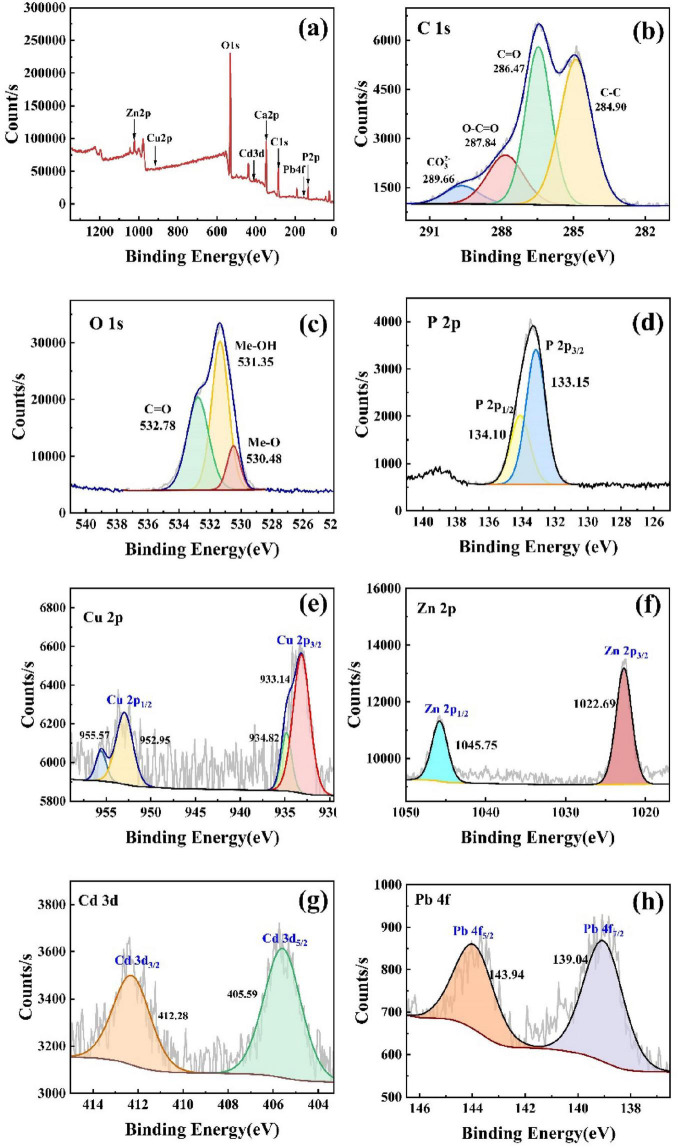
XPS characterization of the precipitates, **(a)** survey spectrum, **(b)** C1s fine spectrum, **(c)** O1s fine spectrum, **(d)** P2p fine spectrum **(e)** Cu2p fine spectrum, **(f)** Zn2p fine spectrum, **(g)** Cd3d fine spectrum, **(h)** Pb4f fine spectrum.

The XPS spectrum of the precipitated product is shown in Figure 7. Among them, Figure 7A was the total spectrum of the sample, and target elements such as C, O, P, Ca, Cu, Zn, Cd, and Pb were detected. It could be seen from Figure 7B that the peak at 284.90 eV was the carbon atom in C–C, and the 286.47 eV was the carbon atom in C = O. These two groups were the primary forms of carbon in the sediment, and the total molar ratio was 78.71%. The two small peaks of 287.84 eV and 289.66 eV were the existence forms of carbon atoms in C–O = C and MeCO_3_, with molar ratios of 15.74 and 5.55%, respectively. Figure 6C was the XPS spectrum of O1s, and three peaks of 530.48, 531.35, and 532.78 eV were fitted, respectively. The first peak at 530.48 eV was the oxygen atom in the direct binding of metal compounds (Farzana et al., 2019). The peak at 531.35 eV originated from the oxygen atom in Me-OH, and the peak at 532.78 eV was caused by carbonate (Bergaoui et al., 2017). Figure 7D showed the XPS peak of P element in the sediment, and a set of peaks of P2p_3/2_ (133.15 eV) and P2p_1/2_ (134.10 eV) were fitted at 133.33 eV. The peak at this position is metal phosphate, which existed as PO_4_^3–^. Figure 7E was the Cu2p_3/2_ and Cu2p_1/2_ peaks of two Cu (II) groups in the 2p orbital of Cu. The two peaks of 933.14 and 934.82 eV at Cu2p_3/2_ represented the presence of CuO and CuCO_3_, accounting for 79.37 and 20.63%, respectively. Figure 7F was the XPS spectrum of Zn in the 2p orbital. By consulting the XPS Handbooks manual, it can be seen that the peak at this point corresponds to the compound Zn_4_(PO_4_)_2_(OH)_2_(H_2_O)_3_. The Cd_3d_ orbital analysis shows that the peak at Cd3d_5/2_ was 405.59 eV, and the Cd in the sediment was divalent. The peak value of lead at Pb4f_7/2_ was 139.04 eV, which existed as divalent and precipitates. The XPS detection found that the target metal valence was consistent with the XRD detection, which indicated that no redox reaction occurred in the system, and the microbial remediation of heavy metals was mainly a biomineralization reaction.

#### 3.6.3 Analysis of sediment structure

**FIGURE 8:**
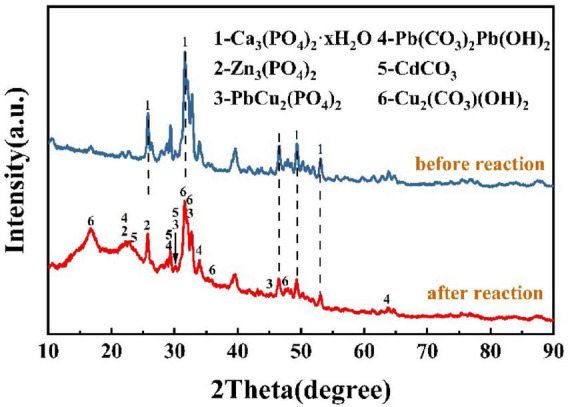
Analysis of sediment structure.

The sediments before and after the reaction were analyzed by X-ray powder diffraction (XRD), and the results of the physical composition of the sediments are shown in Figure 8; the main component of the sediments before the restoration was Ca_3_(PO_4_)_2_-xH_2_O, which was due to the addition of this insoluble substance as one of the components in the culture medium. The XRD pattern peaks during the restoration process were gradually prominent, indicating that the minerals in the precipitated were gradually in an excellent crystalline state. The components of the powder were determined to be Cd_5_(PO_4_)_3_OH, Zn_4_(PO_4_)_2_(OH)_2_(H_2_O)_3_, Cu_3_(PO_4_)(OH)_3_, PbCO_3_, and Cu_3_(OH)_2_(CO_3_)_2_. As the reaction proceeds, the generated stabilization products and the undissolved tricalcium phosphate continue to fill the original loose voids as stabilization continues, and the different stabilization products are wrapped around each other, finally generating a dense biomineralization product, which, in combination with the above findings, suggests that urease-producing bacteria play an important role in the formation of a dense layer. The XRD pattern analysis shows that the products have both phosphate and carbonate. This indicates that after the dissolution of insoluble phosphorus by the PSB, the water-soluble heavy metals combine with soluble phosphate to form new insoluble phosphate precipitates. Under the combined action of the UPB and the PSB, the composite heavy metal precipitated products of phosphate and carbonate were generated from the solution. The dense layer in the precipitate after the reaction consists mainly of carbonates produced by the action of UPB, and the fine particles appearing on the dense layer are Ca_3_(PO_4_)_2_ that has not been dissolved by PSB. The formation of carbonate crystals leads to a narrowing of the pores between particles, and the presence of Ca_3_(PO_4_)_2_ in the medium may provide nucleation sites for the biomineralization. Since biomineralization is a dynamic process in which continuously generated heavy metal phosphates and carbonates are wrapped around each other, and with the external force of the shaker, a dense layer or even a sphere-like morphology is gradually formed.

#### 3.6.4 Changes of microbial morphology before and after stabilization

**FIGURE 9:**
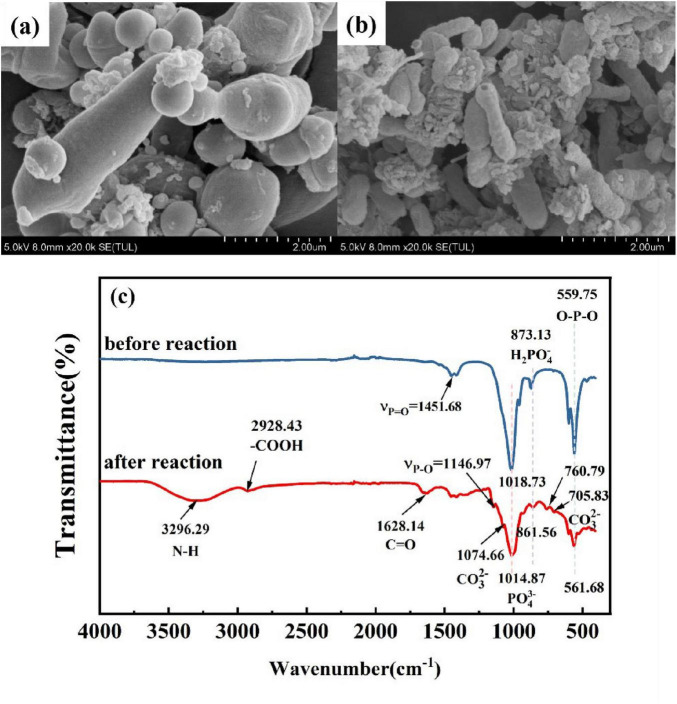
**(A)** SEM image of bacteria before, **(B)** after reaction and **(C)** FT-IR.

The survival state of the bacteria after repair was examined by microscopy. From the SEM images of the bacteria before and after repair (Figure 9), it can be seen that the surface of the bacteria before repair was relatively smooth, and the surface of the bacteria after repair became shrunk, rough and even damaged. More precipitated substances and connective fibrous substances appear. This is because the bacteria produce a large amount of extracellular polymer EPS after receiving heavy metal stress, resulting in more adhesion between cells, which also indicates the adsorption of heavy metals by organisms. Bacterial functional groups play an important role in the biomineralization of heavy metals, such as providing binding sites for heavy metals (Chen et al., 2016), so to explore the role that organisms play in the remediation process, the precipitated products before and after the remediation were subjected to FT-IR infrared analysis, respectively. As can be seen from Figure 9C, functional groups were detected in the system before. After remediation, and as the reaction proceeded, the positions of several peak functional groups stretched, telescoped, or even disappeared, which indicates that the functional groups represented by the above peaks were involved in the reaction and played an essential role in the remediation of heavy metal pollution. The peak value of phosphate in the post-reaction was shifted from 559.75 to 561.68 cm^–1^ and 1018.73 to 1014.87 cm^–1^, respectively, and a significant peak reduction occurred. The spectral peaks representing PO_4_^3–^ were significantly weakened before and after the repair, and the peaks were shifted, indicating that PSB dissolved insoluble phosphate into soluble phosphorus, and part of the soluble phosphorus was absorbed and utilized by the bacteria as an essential element for their life activities, leading to changes in the amount and nature of phosphate in the precipitate (Saranya et al., 2018; Zhou et al., 2023). In addition, other abundant and diverse functional groups (−NH_2_, −OH, -CH, etc.) on the cell surface are also conducive to the binding of heavy metals (Miao et al., 2018). The broad peak at 3296.29 cm^–1^ and the peak at 2928.43 cm^–1^ in the infrared spectra after repair were attributed to the telescopic vibration of the −OH group, −NH_2_ group, and -CH, respectively, which proved that the hydroxyl and amine groups of the bacteria, etc., were involved in the reaction. The appearance of the peaks at 705.83 and 1074.66 cm^–1^ after the stabilization was due to the wobbling vibration of C–O in CO_3_^2–^, while the appearance of the peak at 760.79 cm^–1^ was due to the twisting vibration of C–O in carbonate. After the stabilization, the appearance of the above groups provided an important basis for the participation of functional groups in the biomineralization process, and the appearance of carbonate in the system provided a strong proof for the decomposition of urea by urease-producing bacteria. The porous structure of Ca_3_(PO_4_)_2_ has a large specific surface area, which provides abundant adsorption sites for heavy metals in solution. In summary, the pathways of heavy metals in PSB-UPB stabilization solution include bioprecipitation and biosorption. However, the pathways and specific forms of heavy metal biomigration/transformation in soil still need to be further determined.

### 3.7 The mechanism of mode of heavy metals stabilization by PSB-UPB

**FIGURE 10:**
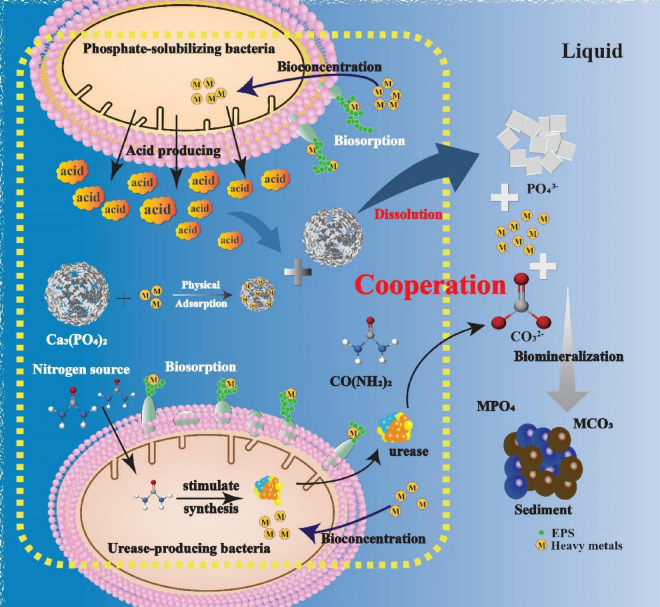
Mechanism mode of heavy metals stabilization by PSB-UPB.

Based on the above characterization, it is clear that the synergistic stabilization of heavy metals by PSB and UPB is the result of a combination of mechanisms. PSB secretes acids through its own metabolism, and the acids react with tricalcium phosphate in the environment to produce free phosphate ions. Phosphate combines with heavy metal ions to form insoluble phosphate precipitates. UPB can form carbonates by breaking down urea to form carbonate precipitates with heavy metals. In addition, heavy metal ions can be adsorbed by tricalcium phosphate through physical adsorption. By XRD and SEM-EDS analyses, it was determined that the main morphology of the curing products was spherical-like precipitates with carbonates and phosphates intertwined. The heavy metals could be stabilized in the form of carbonate and phosphate crystals after the microbial-mediated stabilizing reaction. Further analysis using FTIR revealed that the microorganisms mainly acted in the process of biomineralization and biosorption, and generated carbonate and phosphate species through. In addition, the functional groups such as amide and carboxyl groups produced by the bacteria also actively participated in the bioconcentration process of heavy metals, thus realizing the efficient stabilization of heavy metals. In this synergistic system, three major mechanisms, namely, biomineralization, biosorption and bioconcentration process, play key roles in the stabilization of heavy metals, and the mechanism of mode of heavy metals stabilization by PSB-UPB can be briefly summarized in Figure 10.

## 4 Conclusion

In this study, the pollution control of compound heavy metals was considered from both environmental and ecological aspects, and an environmentally friendly biostabilization bacterial agent was developed by combining urease mineralization technology and dephosphorylated bacteria mineralization technology. The synergistic effect of PSB and UPB can both stabilize the pH of the remediation system and effectively save resources. When the carbon source and nitrogen source were soluble starch and urea, respectively, the average solidification rate of the composite heavy metal solution reached the highest. The stabilization rates of the combined action of PSB and UPB were significantly higher than that of the two microorganisms alone. The solidification products show a dense calcite-like structure, formed mainly by carbonate and phosphate precipitates encapsulated in each other. Specifically, the composite heavy metals in the precipitates are composed of phosphate and carbonate analogues of Cd_5_(PO_4_)_3_OH, Zn_4_(PO_4_)_2_(OH)_2_(H_2_O)_3_, Cu_3_(PO_4_)(OH)_3_, PbCO_3_, and Cu_3_(OH)_2_(CO_3_)_2_. The main ways in which microorganisms such as urease-producing and phosphorus-dissolving bacteria are involved in stabilization are biomineralization, biosorption and bioconcentration.

## Data Availability

The raw data supporting the conclusions of this article will be made available by the authors, without undue reservation.
