# Supplementary figures and images for: Stabilization effect and mechanism of heavy metals by microbial consortium of phosphate-solubilizing bacteria and urease-producing bacteria
Source: Front Microbiol. 2025 Feb 3;16:1525316. doi: 10.3389/fmicb.2025.1525316 (PMC11830682; doi:10.3389/fmicb.2025.1525316)

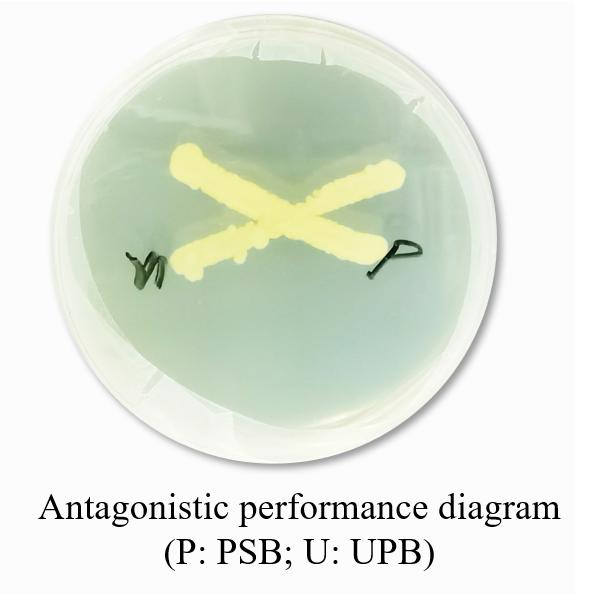

Supplement: Supplementary file 3 [file Image_3.jpeg]
